# Breast cancer detection using sonography in women with mammographically dense breasts

**DOI:** 10.1186/s12880-014-0041-0

**Published:** 2014-12-30

**Authors:** Jimmy Okello, Harriet Kisembo, Sam Bugeza, Moses Galukande

**Affiliations:** Department of Radiology and Radiotherapy, Mulago National Referral and University Teaching Hospital, Kampala, Uganda; Department of Surgery, College of Health Sciences, Makerere University, Kampala, Uganda

**Keywords:** Sonography, Breast cancer, BIRADS, Dense breasts

## Abstract

**Background:**

Mammography, the gold standard for breast cancer screening misses some cancers, especially in women with dense breasts. Breast ultrasonography as a supplementary imaging tool for further evaluation of symptomatic women with mammographically dense breasts may improve the detection of mass lesions otherwise missed at mammography.

The purpose of this study was to determine the incremental breast cancer detection rate using US scanning in symptomatic women with mammographically dense breasts in a resource poor environment.

**Methods:**

A cross sectional descriptive study. Women referred for mammography underwent bilateral breast ultrasound, and mammography for symptom evaluation. The lesions seen by both modalities were described using sonographic BI-RADS lexicon and categorized. Ultrasound guided core biopsies were performed. IRB approval was obtained and all participants provided informed written consent.

**Results:**

In total 148 women with mammographically dense breasts were recruited over six months. The prevalence of breast cancer in symptomatic women with mammographically dense breasts was 22/148 (15%). Mammography detected 16/22 (73%) of these cases and missed 6/22 (27%). The six breast cancer cases missed were correctly diagnosed on breast ultrasonography. Sonographic features typical of breast malignancy were irregular shape, non-parallel orientation, non circumscribed margin, echogenic halo, and increased lesion vascularity (p values < 0.005). Typical sonofeatures of benign mass lesions were: oval shape, parallel orientation and circumscribed margin (p values <0.005).

**Conclusion:**

Breast ultrasound scan as a supplementary imaging tool detected 27% more malignant mass lesions otherwise missed by mammography among these symptomatic women with mammographically dense breasts. We recommend that ultra sound scanning in routine evaluation of symptomatic women with mammographically dense breasts.

## Background

Breast cancer is common in women and a leading cause of cancer mortality in women world wide [1]. In Uganda breast cancer is the third most common cancer in women after cervical cancer and Kaposi’s sarcoma [2]. The incidence of breast cancer in Uganda has nearly tripled from 11:100,000 in 1961 to 31:100,000 in 2006 [3]. Breast cancer cases in sub Saharan Africa present in relatively young women, mostly late in stage III and IV, run an aggressive course and carry a low 5 year survival rate of 39% [4]. Reasons for the early nature of cancer presentation in Uganda is not wholly understood. However multiple factors presumably responsible for this nature of cancer presentation includes genetics, health seeking behaviour and short life span among others.

Mammography as the gold standard imaging method for breast cancer screening in unison with advances in treatment has resulted in reduced breast cancer mortality in the western societies; however this has not been appreciated in resource limited countries like Uganda where access to functioning mammography units and trained personnel is limited. Dense breast tissue has been proven to be the most important inherent limitation of mammography in the diagnosis of breast cancer as some cancers are missed, often requiring ultrasound to complete the breast imaging assesment [5]. In addition dense fibroglandular tissue per se is associated with increased risk of breast cancer and also lowers the sensitivity of mammography to as low as 30-48% [6].

## Methods

### Design

A cross sectional descriptive study.

### Setting

We conducted this study between 1^st^ February to 30^th^ August at Mulago Hospital, the National Referral and a Teaching Hospital for Makerere University located in Kampala, Central Uganda. It has a capacity of 1,500 beds. Mulago Hospital Radiology Department has one functional mammography unit which is uses computed radiography technology to produce digital mammographic images, its open from Monday from Friday and receives patients referred from breast clinics within Mulago as well as the other private hospitals within and outside the city. About 5 diagnostic mammograms were performed daily. The hospital has a functional pathology department offering diagnostic laboratory services managed by experienced pathologists. A female mammographer with a-15-years experience performed the mammograms. The mammograms, breast ultrasound scans and image interpretation as well as US guided core biopsies were performed by a team of consultant radiologists and residents in accordance with the BI-RADS atlas.

### Inclusion criteria

Women 25 years and above with mammographically dense breasts who consented to participate in the study were included. However, women taking hormone replacement therapy were excluded.

### Sampling and data collection

Women referred for mammography were x-rayed in accordance with the cut off age spelt in the Uganda breast cancer clinical guidelines. Those with mammographically dense breasts were consecutively recruited upon obtaining an informed written consent to participate in the study.

Data was collected using a pre-coded and pre tested questionnaire. Study variables included;

Socio-demographic data such as Age, gender, menopausal status, indication for mammography, mass lesion visibility on mammogram, Sonographic BI-RADS descriptors, BI-RADS final assessment categorization as well as histological diagnosis.

The mammograms were performed using Phillips Mammogram diagnost UC model 2000 with a dual focal spot 0.3/0.1 mm acceptable for both diagnostic and screening purpose. 18 × 24 cm imaging plates with a single intensifying screen for computed radiography. Philips Computed Radiography computer system with its laser printer.

A Philips HD7 2009 model manufactured by Philips and Neusoft Medical systems Co. Ltd, Shenyang, China, with a 7. 5 to12 MHz broad band linear probe were used to scan the patients.

US-guided core biopsies were performed using needle gauze 14.

Standard mammographic views (Mediolateral oblique and Craniocaudal views) were performed in accordance with the international atomic energy agency (IAEA) human health series [18]. The imaging plates were then processed using the computed radiography system inorder to print CR mammograms. The mammograms were subsequently viewed systematically on a dedicated mammographic film viewer box by a team that consisted of the Consultant Radiologists and radiology residents. The mammographic breast density category was categorized according to the ACR BI-RADS atlas breast density categories recorded as 1,2,3 or 4. The final conclusion reached on consensus by the team on the breast density category and final mammographic diagnosis was documented on the questionnaire by the principle investigator. The BI-RADS atlas was available to the team and was helpful in sorting out interobserver disagreements that arose during interpretation.

Bilateral whole breast ultrasound scan was performed on all the study participants for atleast one of the following reasons;

Further evaluation of mammographically dense breast tissue inorder to complete breast image work up or ultrasound guided biopsy of the detected breast lesions when indicated.

The process of breast sonographic examination was explained to the patient before performing it. While observing privacy in the examination room all patients had to change to a clean examination gown with adequate exposure of chest wall. A chaperone was present during the procedure.

The patients were positioned lying supine oblique on a clean examination bed with the ipsilateral hand extended above the head to stabilize and flatten breast against the chest wall. This positioning was done for both breasts.

An acoustic gel was applied on the breast prior to scanning using the linear probe.

Both breasts were systematically scanned with overlapping scans in a radial and antiradial pattern from the nipple to the periphery. The retroareolar region including both axillae were scanned separately with angled probe views to ensure the complete coverage of all breast tissue. The images were saved as a soft copy in the US machine and copied to DVD blanks. A hard copy print on thermal paper was also made for some patients.

A report on breast sonographic findings was written down in accordance with the BI-RADS US lexicon adapted from the American college of radiology for standardization and given to the study participants.

Sonographs were blinded to the mammograph results.

A total of 45 US guided core biopsies were performed on breast mass lesions categorized as BI-RADS final assessment categories 4, 5 as well as some category 3 cases.

### Biopsy procedure

An informed consent for biopsy was obtained after thorough explanation of the procedure to the study patients.

Both breasts including the axillae were systematically scanned with overlapping scans in a radial and antiradial pattern until a mass lesion was localized.

Under local anaesthesia and aseptic technique ultrasound guided core needle biopsy of solid breast masses were performed by a standard free hand technique using a disposable automated 14-gauge needle with a 22 mm throw. The breast tissue sampled was put in a biopsy bottle containing formalin and taken for histopathological analysis. At least 3 biopsy samples was taken from each lesion for diagnostic adequacy of the sample.

In case the lesions were sonographically similar then only one of the most prominent lesions was biopsied.

If the lesions are sonographically differing in appearance then at least two of the lesions were biopsied. Ultrasound guided fine-needle aspiration biopsy was done for complex cysts or masses, sample was air dried for 5 seconds prior to fixing it on the slide using ethanol 95% solution.

We took the biopsy samples to pathology laboratory for cytopathological analysis.

### Data management

Questionnaires were checked for completeness. Data was entered into the computer using EPI DATA version 3.1. It was exported to STATA version 2013 for analysis. Statistical methods to analyze the data included univariate, bivariate analyses. Categorical and nominal variables were summarized using proportions, frequency tables, pie chart, and histograms.

### Quality control

Quality control was ensured using the following measures:

The questionnaire was pre tested before commencement of the study to ascertain if the required information could be obtained using the specified questions.

Breast imaging interpretation was performed by a team, with BI-RADS atlas [12] available for reference to ensure correct interpretation was made.

### Ethical consideration

Approval was obtained from Makerere University College of Health Sciences and IRB of Mulago Hospital.

Patient confidentiality was ensured.

## Results

**Figure 1:**
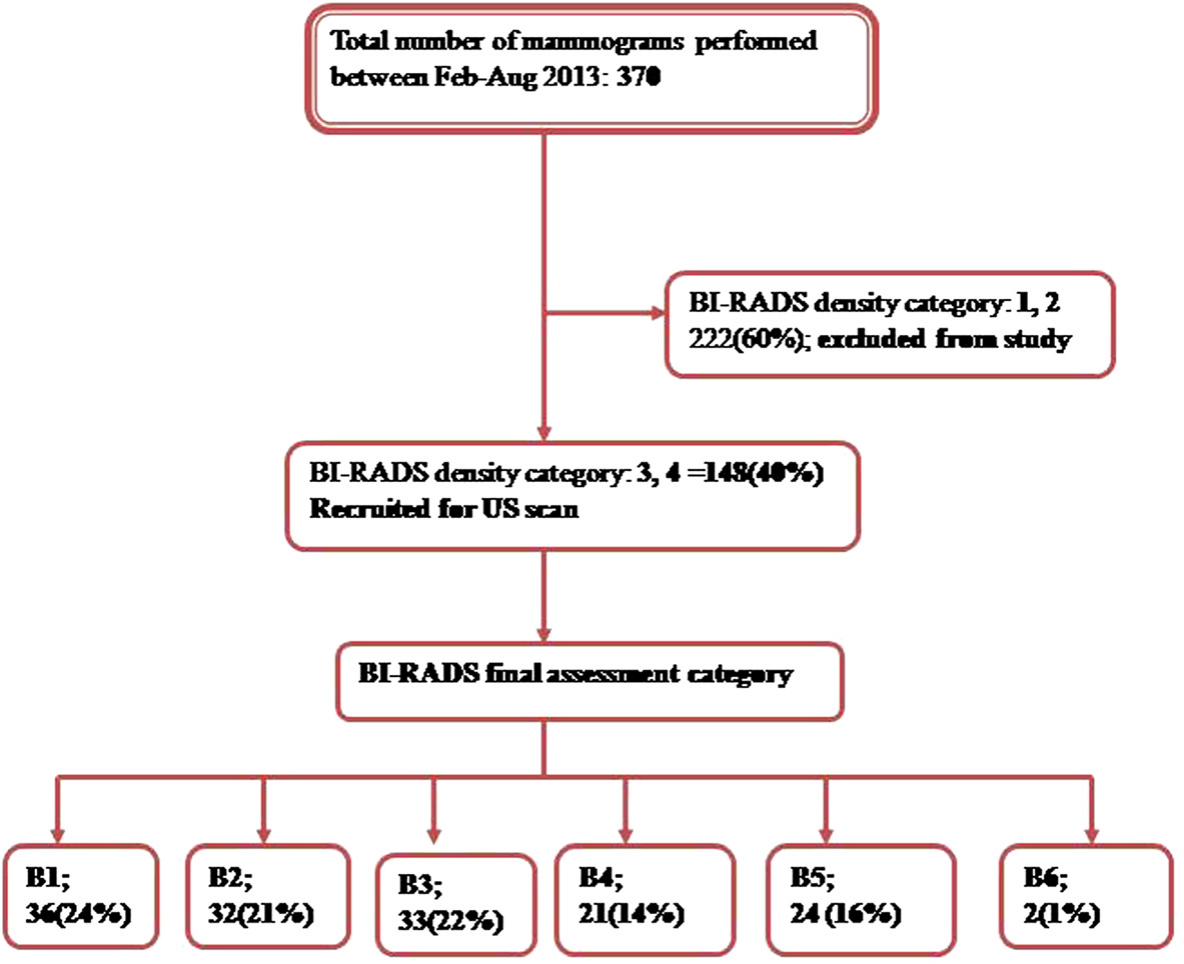
**Study flow chart showing participants recruitment and outcomes.**

Out of the total 370 mammograms performed, 148 were categorized as BI-RADS density category 3 or 4 (mammographically dense breast tissues) and all underwent bilateral breast ultrasound scan. A total of 111 lesions were detected and described using the BI-RADS lexicon and final assessment categorization was made. US guided biopsy was done for 43 patients with BI-RADS final assessment category 4 or 5 lesions. 2 patients with breast masses categorized as BI-RADS 4 were not biopsied as they did not return for their biopsy appointment (see Figure 1).

The characteristics of lesions missed on mammography are indicated in Table 1.

**Table 1:** **Characteristics of the mammographically missed cancer cases**

**Cases**	**Age**	**Complaint**	**BI-RADS density**	**Tumor size (mm)**	**US BI-RADS categorization**
1	45	Pain	3	10	BI-RADS 4
2	31	Lump	4	12	BI-RADS 5
3	28	Lump	4	22	BI-RADS 4
4	35	Lump	4	15	BI-RADS 5
5	38	Discomfort	3	12	BI-RADS 4
6	29	Painful lump	4	9	BI-RADS 4

Several BIRADS sonographic descriptors were used and included shape, orientation margins, boundaries, echo texture, posterior acoustic feature, surrounding tissues, calcification, lesion vascularity size and lymph nodes. Shape, orientation, margins, boarders and vascularity differentiated between benign and malignant, see Table 2.

**Table 2:** **Frequency of BI-RADS sonographic descriptors and its correlation with benign versus malignant outcome**

**BI-RADS sonographic descriptors**	**Frequency: n (%)**	**Malignant outcome: n (%)**	**Benign outcome: n (%)**	**P-Value**
**Shape**				0.004
Oval	15 (13.9)	2 (13.3)	13 (86.7)	
Round	50 (46.3)	25 (50.0)	25 (50.0)	
Irregular	43 (39.8)	41 (95.4)	2 (4.6)	
**Orientation**				0.001
Parallel to skin	67 (62)	13 (19.4)	54 (80.6)	
Not parallel	41 (38)	40 (97.6)	1 (2.4)	
**Margin**				<0.001
Circumscribed	53 (49.1)	5 (9.4)	48 (90.6)	
Non circumscribed	55 (50.9)	52 (94.5)	3 (5.5)	
**Lesion boundary**				0.004
Abrupt interface	80 (74.1)	53 (66.3)	27 (33.7)	
Echogenic halo	28 (25.9)	26 (92.8)	2 (7.2)	
**Echo texture**				0.448
Anechoic	14 (13.0)	7 (50.0)	7 (50.0)	
Hypoechoic	74 (68.5)	53 (71.6)	21 (28.4)	
Complex	17 (15.7)	16 (94.1)	1 (5.9)	
Hyperechoic	3 (2.8)	0 (0.0)	3 (100)	
**Posterior acoustic feature**				0.458
No posterior feature	40 (37.0)	24 (60.0)	16 (40.0)	
Enhancement	32 (29.6)	21 (65.6)	11 (34.4)	
Shadowing	18 (16.7)	17 (94.4)	1 (5.6)	
Combined	18 (16.7)	16 (88.9)	2 (11.1)	
**Surrounding tissues**				0.448
Normal	65 (60.2)	15 (23.1)	50 (76.9)	
Architectural distortion	20 (18.5)	19 (95.0)	1 (5.0)	
Skin thickening	11 (10.2)	7 (63.6)	4 (36.4)	
Subcutaneous oedema	11 (10.2)	8 (72.7)	3 (27.3)	
Nipple retraction	1 (0.9)	1 (100.0)	0 (0.0)	
**Calcification**				0.406
Micro calcification	35 (32.4)	34 (97.1)	1 (2.9)	
Macro calcification	2 (1.9)	2 (100.0)	0 (0.0)	
No calcification	71 (65.7)	43 (60.6)	28 (39.4)	
**Lesion vascularity**				0.002
Increased vascularity in mass	41 (37.9)	37 (90.2)	4 (9.8)	
Avascular	56 (51.9)	29 (51.8)	27 (48.2)	
Increased surrounding vascularity	11 (10.2)	9 (81.8)	2 (18.2)	
**Widest diameter of mass**				NA
<1 cm	6 (5.6)	1 (16.7)	5 (83.3)	
1-2.5 cm	44 (40.7)	27 (61.4)	17 (38.6)	
>2.5 cm	58 (53.7)	52 (89.7)	6 (10.3)	
**Abnormal lymph nodes**				NA
Present	73 (67.6)	60 (82.2)	13 (17.8)	
Absent	35 (32.4)	19 (54.3)	16 (45.7)	

Note: Numbers in parentheses are percentages of each group.

Frequency: Number of times this US feature was reported to be present.

Malignant outcome = number of masses reported to have this feature that were considered malignant.

Benign outcome = number of masses reported to have this feature that were benign.

NA = Not applicable as the US feature is not a recognized BI-RADS descriptor according to ACR.

**Figure 2:**
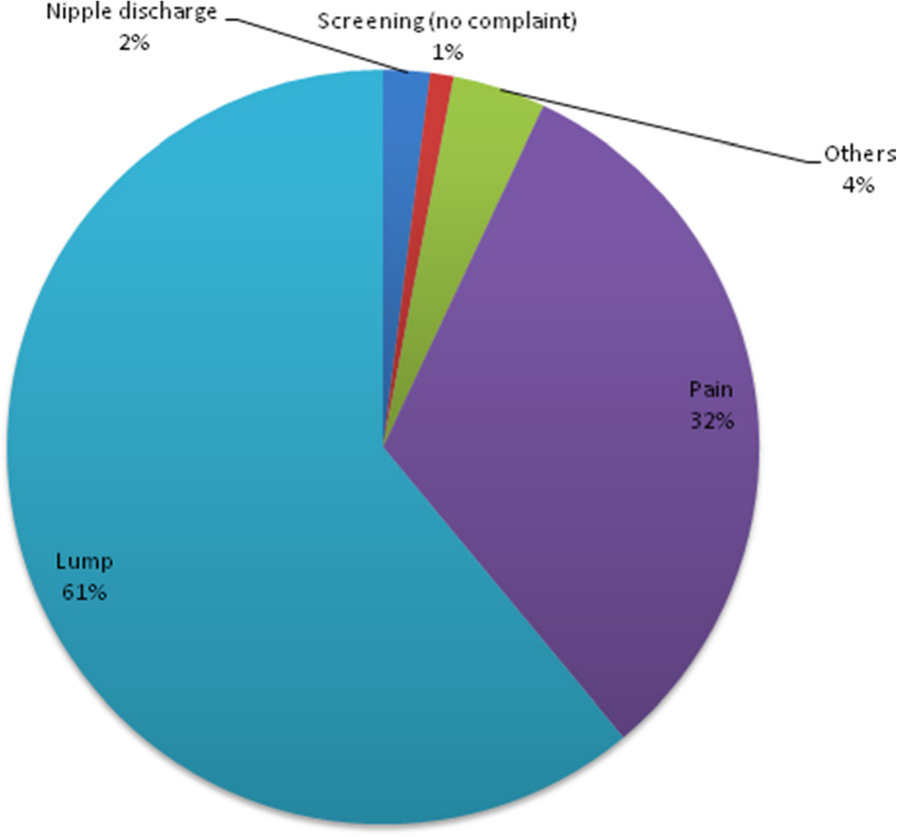
**The main presenting complaint of the study patients.**

In Figure 2 we show the presenting complaints; a lump being the most prevalent, followed by breast pain.

**Figure 3:**
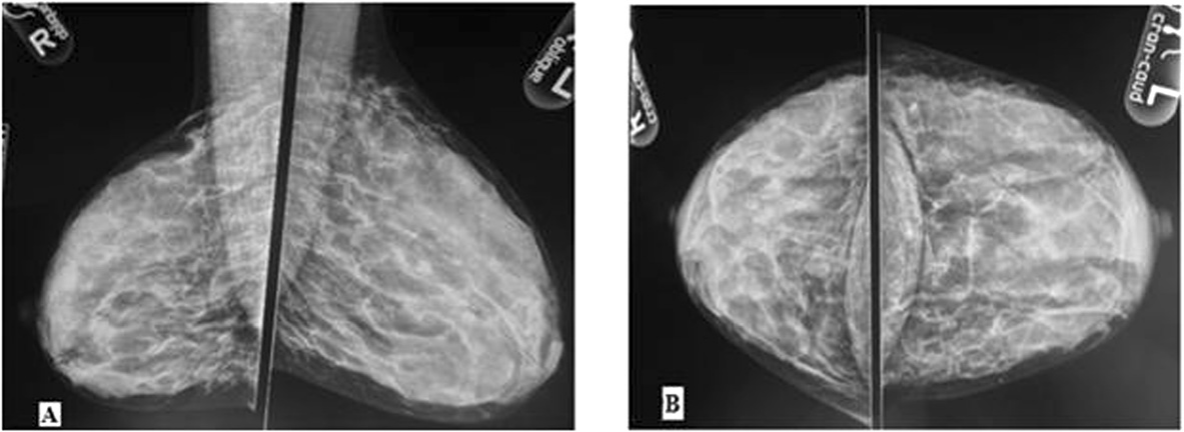
**Mammographic films of a 31-year old woman who presented with a palpable left breast lump for 4 months, A: Oblique views B: craniocaudal views.**

Figure 3A and B mammogram show a sample of a BIRADS 3 density. The mammogram shows BI-RADS density category 3 and no focal mass is demonstrable.

**Figure 4:**
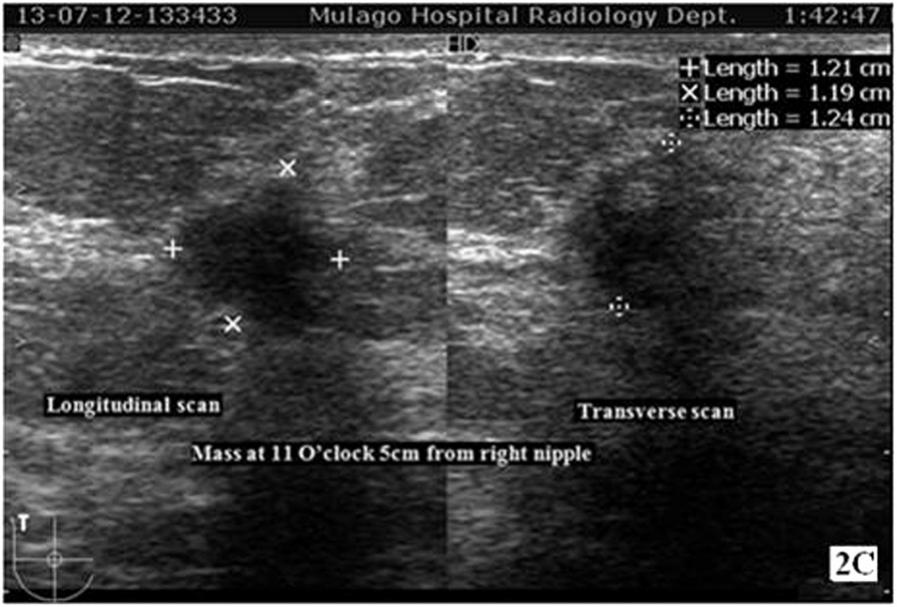
**Sonogram shows a solid hypoechoic mass which is irregular in shape, has angular margin with surrounding echogenic halo at 11 O’clock 5 cm from right nipple.**

Figure 4 shows a sonogram of a solid mass with descriptors suggesting a malignancy is shown.

US guided biopsy revealed a poorly differentiated infiltrative lobular carcinoma.

Majority of the women were symptomatic with palpable breast lump. Only two women came for breast cancer screening.

Diagnostic performance of US scan and mammogram is described and shown in Table 3.

**Table 3:** **Diagnostic performance of mammography versus ultrasound in visualizing mass lesion in women with mammographically dense breast tissue**

**Mass (lesion) seen**	**Mammogram**	**Ultrasound**	**Mammographic findings**	**Ultrasound plus Mammographic findings; (BI-RADS final assessment)**
			**Conclusive diagnosis**	**Conclusive diagnosis**
	**Frequency; N (%)**	**Frequency; N (%)**	**Frequency; N (%)**	**Frequency; N (%)**
Yes	69 (62.2)	110 (99.1)	71 (64)	111 (100)
No	42 (37.8)	1 (0.9)	40 (36)	0 (0)
**Total**	111	100	111	100

The most frequent histological type was ductal carcinoma 54% followed by lobular carcinoma. The most common benign lesions were firoadenomas (see Table 4).

**Table 4:** **Breast lump histological diagnoses**

**Breast cancer cases**	
**Histological type**	**Frequency; N (%)**
Invasive ductal carcinoma	12 (54)
Infiltrating lobular carcinoma	2 (9)
Adeno carcinoma	6 (27)
Lymphoma	1 (5)
Alveolar rhabdomyosarcoma	1 (5)
Total	22 (100)
**Benign breast conditions**	
Fibro adenoma	7 (33)
Cystic mastopathy	4 (19)
Chronic inflammation	5 (24)
Sclerosis adenosis	1 (5)
Other benign conditions	4 (19)
Total (benign conditions)	21 (100)

In all 22 breast cancer cases were correctly diagnosed using sonography and occurred in relatively young women averaging to 41 years in age (age range; 28-59 years), see Table 4.

## Discussion

We set to investigate the incremental breast cancer detection rate of breast ultrasonography as a supplemental imaging tool in evaluation of symptomatic women with dense breasts (BIRADS 3 & 4). We found that US Scan detected 27% more malignant lesions than mammography did. The odds of mammography missing a malignant breast lesion in dense breasted women were 1 in 4. The missed lesions were likely to be 10 mm or less in their widest diameter. Reasons for missing these malignant mass lesions could be the dense tissues obscuring visualization of those small sized tumors at mammography. However all the missed lesions were detected at US scan which is not limited by breast density. These findings are important because small lesions (less than 20 mm) are mostly early breast cancer lesions and are amenable to curative treatment. In addition ultrasound is more accessible than mammogram in our environment, therefore becomes an attractive supplement to mammography [3].

The BI-RADS sonographic lexicon was helpful in distinguishing benign from malignant solid breast masses with typical signs of malignancy being irregular shape, anti-parallel orientation, non circumscribed margin, echogenic halo, and increased lesion vascularity. Typical signs of benignity were oval shape and circumscribed margin (p < 0.005).

Mass echo texture, posterior acoustic features and surrounding tissues of a mass as well as presence or absence of lymph nodes were not reliable in differentiating between benign and malignant mass lesions (P > 0.005).

A total malignancy rate of 14.9% (22/148) is three fold higher compared to a previous study by Paulo et al. which showed a prevalence of 4.2% among symptomatic patients with dense mammograms [16].

Breast cancer occurred in relatively young women averaging to 41 years in age (age range; 28-59years). This finding is in keeping with literature which shows that more than half of women between 25 and 49 years of age have dense breasts with more cancer risks, as do approximately 29% of women older than 50 years [4,5].

Nearly all the women were symptomatic; 99% (n = 146) and only two women (1%) came in for breast cancer assessment.

Tumors and glandular tissue have a similar dense appearance on mammography, making it difficult to distinguish metabolically active normal breast tissue from cancer. As a result, the performance of mammography in women with high breast density is poor [3,6-10]. The relative availability of ultrasound makes it an attractive imaging modality for evaluating women for breast cancer in resource-limited settings where other modalities like MRI are not readily available.

### Correlation of sonographic features with benign versus malignant outcome

A standardized lexicon for sonography was developed in 2003 by the ACR in light of the increasing use of sonography in clinical practice. Like its mammographic counterpart, the sonographic BI-RADS lexicon was intended to provide a unified language for sonographic reporting and research and to avoid ambiguity in the communication and teaching of sonographic interpretation [12]. This lexicon helps the radiologist in describing sonographic features and defining the final assessment category that is associated with the most appropriate clinical management of the case.

Sonographic BI-RADS descriptors that most reliably characterized a mass as malignant study include an irregular shape, non circumscribed margin, non parallel orientation, echogenic halo around a mass, and increased vascularity within the mass.

In this study, Sonographic BI-RADS descriptors highly predictive of benignity of a mass were circumscribed margin; 90.6% (48/53), parallel orientation; 80.6% (54/67), and oval shape; 86.7 (13/15). Bi-variate analysis of these descriptors (margin, shape, orientation, lesion boundary and vascularity chosen were significantly reliable in differentiating malignant and benign (*p* < 0.005).

This is in conformity with prior study findings that, these BI-RADS descriptors represent an abnormal disease process in the breasts [11,13].

A circumscribed margin is well defined or sharp, with an abrupt transition between the lesion and surrounding tissue usually predicts a benign outcome.

As in mammography, sonographic evidence of non circumscribed margins (which includes one of these options: spiculated, angular, microlobulated and indistinct margins) suggests infiltrating growth of the lesion into the surrounding tissue which is most times predictive of a malignant outcome.

Irregular shapes indicate inconsistent growth and advancement of the lesion edge which usually predicts a malignant outcome while for a benign mass usually takes on an oval or a round shape.

A parallel orientation is when the long axis of lesion parallels the skin line (“wider than tall” or horizontal) whereas a non-parallel orientation refers to a long axis, not oriented along the skin line (“taller than wide” or vertical, includes round masses).

Non-parallel orientation on sonography may suggest spread of the lesion through tissue-plane boundaries, a characteristic which is more likely to be associated with malignant lesions. In contrast, circumscribed margins and oval shapes represent smooth uniform growth without involvement of surrounding tissue and are associated more with a benign lesion. Similarly, parallel orientation suggesting containment in one tissue plane and indicative of a benign process.

Lesion boundary refers to the demarcation between the mass lesion and surrounding tissues. Identification of surrounding echogenic halo was a reliable BI-RADS descriptor in predicting a malignant outcome in this study. This agrees with previous studies which showed that identification of surrounding tissue effects had a high predictive value for malignancy, suggesting that recognition of such features could be helpful in the final assessment categorization at ultrasonography [12,13].

Increased vascularity within a mass lesion was a reliable BI-RADS descriptor of malignancy, a finding that agrees with literature.

Mass echo texture, posterior acoustic features and surrounding tissues of a mass as well as presence or absence of lymph nodes were not reliable in differentiating between benign and malignant mass lesions in this study.

The most common malignancy was invasive ductal carcinoma which accounted for 54.5% (n = 12) of histopathological examination results obtained. This histopathological finding confers with previous studies [14,15]. The cancer yield is comparable to the expected rate of malignancy in BI-RADS final assessment categories. Benign breast disease was also not uncommon with fibroadenoma being the most commonly encountered histological diagnosis.

Several factors limit the use of mammography in breast cancer detection in Uganda. First, breast cancer peaks in younger women who more frequently have denser breasts and, therefore the sensitivity of mammography is reduced. Second, younger women [17] are more sensitive to ionizing radiation. Several other benefits to using ultrasound include its relatively cheaper costs, its availability in resource-limited countries, no limitation by fibroglandular breast composition, its ability to be used for image guided-biopsies with relatively little additional training and equipment, and its portability [7-9]. For these reasons, ultrasound is an attractive imaging modality for evaluating women for breast cancer in a resource-limited country such as Uganda. According to the latest BI-RADS atlas, it is mandatory that a mammographically dense breast tissue needs further additional imaging evaluation in order for the interpreting radiologist to make a conclusive radiological diagnosis. This further imaging evaluation is most times completed using high frequency breast ultrasound and rarely requiring MRI scan [10]. The are only five mammography machines which are inequitably distributed in Uganda compared to over 100 high frequency range ultrasound machines capable of breast sonography. This study was carried out with the aim of evaluating the use of bilateral whole breast ultrasound scan as an adjunctive imaging tool to detect cancer in women with dense breasts at Mulago hospital. Breast mass lesions detected were described according to the BI-RADS lexicon and final assessment categorization made.

### Study limitations

This was a cross sectional descriptive study and so short term interval follow up of breast mass lesions categorized as benign and probably benign (BI-RADS 2 and BI-RADS 3 respectively) was not carried out to ascertain radiological and clinical stability of these mass lesions. Not all benign breast lesions were biopsied hence sensitivity and specificity of breast ultrasound could not be calculated.

## Conclusion

Breast ultrasound scan resulted in significant incremental breast cancer detection rate (of 27%) among symptomatic women with mammographically dense breast tissue. We recommend that breast ultrasound scan should routinely be done in mammographically dense breasts (BI-RADS density category 3 and 4) in resource limited settings.
